# The change in blood glucose levels in tuberculosis patients before and during anti-tuberculosis treatment in China

**DOI:** 10.1080/16549716.2017.1289737

**Published:** 2017-05-04

**Authors:** Yan Lin, Yanli Yuan, Xin Zhao, Jianmu Liu, Linxi Qiu, Xiaoxin He, Yunlong Bai, Bo Li, Zhong Zeng, Guangxu Yang, Xu Meng, Chunyu Yuan, Hailun Zheng, Wanli Kang, Anthony D. Harries

**Affiliations:** ^a^ China Office, International Union Against Tuberculosis and Lung Disease, Beijing, China; ^b^ Director Office, Jilin Provincial Academy of Tuberculosis Control and Prevention, Changchun, China; ^c^ Department of Laboratory Medicine, Beijing Hospital, Beijing, China; ^d^ TB Department, Jilin City Tuberculosis Institute, Jilin, China; ^e^ Director Office, Jiangxi Provincial Institute of Tuberculosis Control and Prevention, Nanchang, China; ^f^ Director Office, Beijing Tuberculosis Institute, Beijing, China; ^g^ TB Department, Ganzhou No. 5 Hospital, Ganzhou, China; ^h^ Office of TB Care and Prevention, Changchun Infectious Disease Hospital, Changchun, China; ^i^ TB Section, Ganzhou CDC, Ganzhou, China; ^j^ Director Office, Shuangyang District Tuberculosis Institute, Changchun, China; ^k^ Beijing Chest Hospital, Capital Medical University, Beijing, China; ^l^ Department of Epidemiology, Beijing Tuberculosis and Thoracic Tumor Research Institute, Beijing, China; ^m^ Department of TB and HIV, International Union Against Tuberculosis and Lung Diseases, Paris, France; ^n^ Department of TB and HIV, London School of Hygiene and Tropical Medicine, London, UK

**Keywords:** Blood sugar, tuberculosis, China

## Abstract

**Objective**: We aimed to observe (i) changes in fasting blood glucose (FBG) in tuberculosis (TB) patients before and during anti-TB treatment, (ii) whether FBG levels were stable or unstable and (iii) baseline characteristics associated with an unstable FBG.

**Method**: TB patients consecutively attended six clinics or hospitals. FBG measurements were made at months 0, 2 and 6. Data analysis was performed using the chi-square test and multivariate logistic regression.

**Results**: Of 232 patients without diabetes mellitus (DM) whose initial FBG < 6.1 mmol/L, over 90% maintained FBG < 6.1 mmol/L during treatment and no patient developed DM. Of 17 patients without DM and initial FBG between 6.1 and 6.9 mmol/L, over half had FBG < 6.1 mmol/L during treatment and no patient had DM at the end of treatment. Eight DM patients with already known DM had their FBG controlled at < 7.0 mmol/L during treatment. There were 13 DM patients newly diagnosed with FBG ≥ 7.0 mmol/L, and 69% continued to have FBG ≥ 7.0 mmol/L. After adjustment for confounding, the odds for an unstable FBG were higher for HIV-positive status, already having DM, smoking and coming to hospitals rather than clinics.

**Conclusion**: TB patients who do not have DM based on FBG measurements do not develop DM during anti-TB treatment. Those newly diagnosed with DM on screening in general maintain their DM status with high FBG and need to be better managed.

## Background

Despite efforts for the last 25 years from the global community to control tuberculosis (TB), the disease remains a major global public health threat. In 2014, an estimated 9.6 million people developed active TB and 1.5 million people were estimated to have died from the disease [1]. In the last decade, the world has also witnessed an escalating epidemic of diabetes mellitus (DM) which has arisen as a consequence of population growth, aging, urbanization and lifestyle changes. Available data suggest that an estimated 415 million people worldwide live with DM and another 318 million people have impaired glucose tolerance, which if left unchecked can progress to DM. By 2040 these numbers are predicted to grow to 642 million and 481 million, respectively [2].

People with DM have a significantly higher risk of developing active TB which is 2–3 times higher than in those with no diabetes [3]. DM patients with TB are also reported to have worse treatment outcomes compared with patients without DM, with delays in sputum culture conversion, an increased risk of failure or death during anti-TB treatment and an increased risk of recurrent disease after successful completion of anti-TB treatment [4–6].

To address this progressively worsening health problem in low- and middle-income countries and the linkage between the two diseases, the World Health Organization (WHO) and the International Union Against Tuberculosis and Lung Disease (the Union) launched a ‘Collaborative Framework for Care and Control of Diabetes and Tuberculosis’ in 2011, with one of the important recommendations being the implementation of bi-directional screening of the two diseases within the routine health services [7].

In the meantime, The Union, together with national authorities, implemented the first bi-directional screening of DM and TB in 6 TB clinics and 5 DM clinics within the routine health services across China. Bi-directional screening was found to be feasible with two published studies describing the implementation, the results and the challenges of screening DM and TB within routine health care settings [8,9].

During the screening of TB patients for DM, one of the important issues with the process, which is undertaken soon after TB registration and at the start of anti-TB treatment, is that TB may induce infection-related hyperglycaemia [9–11]. A small-scale study in Nigeria, assessing blood glucose tolerance at multiple points during the course of anti-TB treatment, also reported that the prevalence of abnormal results decreased over time [12], leading to the questions of when is the best time to take blood glucose measurements and whether one blood test is enough to diagnose or rule out DM for the majority of TB patients. To help answer these questions, we conducted an observational cohort study to understand if blood glucose levels were stable or fluctuated during the course of anti-TB treatment in multiple settings in different provinces of China. This paper reports the findings from this study.

## Methods

### Design

This was a prospective cohort observational study carried out at six TB clinics and hospitals within routine health services in three provinces of China. The total sample size was calculated using a 2-sided significance level of 0.05 and a power of 0.8. We assumed that 8% of TB patients at TB clinics would have unstable FBG, but the proportion of TB patients with unstable FBG at hospital was in fact 20%. Therefore, the minimum required sample size was 221. Taking into account a loss to follow-up of about 20% or patients not presenting within ±7 days at the end of month 2 or the end of month 6 of anti-TB treatment, we needed to recruit at least 266 TB patients.

### Setting and sites

The experience of screening TB patients for DM in 2011–2012 led to a decision that the study should be carried out in settings where different types of TB patient could be included. Therefore, four TB clinics and two TB hospitals across mainland China were selected. These were: Changchun Communicable Disease Hospital, Jilin City TB Institute, Shuang Yang District TB Institute, Ganzhou No. 5 Hospital, Beijing TB Institute and Yudu County TB Institute. Selection of the clinics and hospitals was based on broad geographical coverage, a sufficient number of TB patients stratified by different types of disease who had been registered 1 year previously, availability of TB and DM diagnostic and treatment facilities and willingness of the staff to participate in this study.

### Patients

All patients who were consecutively diagnosed and registered with TB from February to July 2015 were included in the study.

### Anti-TB treatment

All TB patients were registered, treated and managed according to the guidelines of the China National TB Control Program [13]. Treatment regimens and anti-TB drug formulations were administered in accordance with those recommended by WHO and in line with National Tuberculosis Program (NTP) guidelines [14]. The standardized treatment regimen consists of 2 months of thrice-weekly isoniazid, rifampicin, pyrazinamide and ethambutol followed by 4 months of thrice-weekly isoniazid and rifampicin.

### Blood sugar measurement for TB patients

Blood samples were collected for FBG in all patients after an overnight fast of at least 10 hours. FBG measurements were in line with national guidelines which stipulate that a FBG measurement is carried out using venous plasma and a biochemical analyzer with cut-off thresholds based on those recommended by the WHO [15]. In brief, a fasting blood glucose (FBG) ≥ 7.0 mmol/L (126 mg/dl) indicates DM; a FBG of 6.1–6.9 mmol/L (110 mg/dl to less than 126 mg/dl) indicates impaired glucose tolerance; a FBG < 6.1 mmol/L (110 mg /dl) is normal. For every newly diagnosed and registered TB patient, we took the first blood sample for FBG immediately after the TB diagnosis; and this was followed by FBGs within ±7 days of the end of month 2 and the end of month 6 of anti-TB treatment, respectively. For any patient who was not fasting at the first visit, an additional visit was scheduled for FBG to be performed on the following day.

### Data collection and recording

A standardized questionnaire was developed for data collection focusing on demographic characteristics, types of TB, symptoms, complications and cigarette smoking status. Patients were diagnosed with DM either as a result of this being known (diagnosed at any time by a registered medical institution and documented in the clinic notes) or as a result of FBG ≥ 7.0 mmol/L being found at the time of TB registration or at the time of TB diagnosis from another health facility. The results of the FBG measurements at the different time periods were also recorded in the same questionnaire. The questionnaire was compiled by TB clinic staff and reviewed by Union staff during the monitoring visit and before data analysis.

### Quality assurance

#### Blood sample

Blood samples for FBG at the six TB clinics/hospitals were all from venous plasma: no capillary blood tests were done.

#### Biochemical analyser

The type of biochemical analyser used at the four TB clinics was Mairui BS-300, BS-320 or BS-350, produced in Shenzhen, China; the analyser type at the two hospitals was a Hitachi-7100. National Reference Laboratory staff ensured that the different biochemical analysers gave consistent and accurate results through external quality assurance.

#### Field monitoring

Supervision and site visits were carried out by staff of the Union and provincial TB staff during the period of the study.

### Data analysis and statistics

Individual patient data were received and cross-checked by staff of the Union and provincial TB staff, and were then double-entered into an *Excel* file and analysed. TB patients were categorized into having stable FBG (defined as a variation between baseline FBG and month 2 and/or month 6 FBG of ≤ 1.0 mmol/L) or unstable FBG (defined as a variation between baseline FBG and month 2 and/or month 6 FBG of > 1.0 mmol/L). Comparisons of baseline characteristics between TB patients with stable or unstable FBG were carried out using the chi-square test with odds ratios (O.Rs) and their 95% confidence intervals. Levels of significance were set at 5%. We selected variables with unadjusted O.Rs for which the *P*-value was < 0.2 and included these in the multivariate logistic regression model.

## Results

There were 303 TB patients consecutively registered in this study. Of these, 15 were lost and 18 missed the accepted timing of FBG which should have been done within ±7 days of the end of month 2 or the end of month 6 of anti-TB treatment. Therefore 270 TB patients were eligible for this study. This included 68 with smear-positive pulmonary TB, 187 with smear-negative pulmonary TB and 15 with extra pulmonary TB (EPTB). There were 179 males and 91 females, aged from 10 years to 88 years old with an average age of 42.1 years. Of the 270 TB patients, 21 had DM (8 were already known to have DM and 13 were newly diagnosed as having DM) and 249 had no DM. The distribution of FBG levels for those without DM was as follows: 232 persons had their FBG < 6.1 mmol/L and 17 persons had their FBG between 6.1 and 6.9 mmol/L; no person had FBG ≥ 7.0 mmol/L. Of the 21 patients with DM, the FBG level at the time of registration was as follows: 5 persons had their FBG < 6.1 mmol/L, 3 persons had their FBG between 6.1 and 6.9 mmol/L and 13 had their FBG ≥ 7.0 mmol/L.

Table 1(A–D) shows the number of TB patients with different FBG levels at month 2 and month 6, stratified by those without DM and those with DM. For those without DM whose initial FBG < 6.1 mmol/L (Table 1A), over 90% had their FBG < 6.1 mmol/L in month 2 and month 6, with 4.7% and 7.3% moving up into the FBG range of 6.1–6.9 mmol/L in month 2 and month 6, respectively. Of the 17 patients without DM whose initial FBG was between 6.1 and 6.9 mmol/L (Table 1B), over half moved to an FBG < 6.1 mmol/L in months 2 and 6. Three of the patients moved to FBG ≥ 7.0 mmol/L at month 2, but all moved down in month 6. For the eight patients with already known DM, blood glucose levels varied at month 2 or 6 with over half of the patients at each time period having FBG < 7.0 mmol/L (Table 1C). For the 13 patients with newly diagnosed DM and FBG ≥ 7.0 mmol/L at month 0 (Table 1D), most continued to have FBG ≥ 7.0 mmol/L with only one patient (the same patient) having a normal FBG at month 2 and month 6, respectively.

**Table 1.** Fasting blood glucose levels at month 2 and month 6 in TB patients, stratified by those without DM and those already diagnosed or newly diagnosed with DM.

	Month 0	Month 2	Month 6
**A: Fasting blood glucose levels at month 2 and month 6 in TB patients without DM whose fasting blood glucose < 6.1 mmol/L at the time of registration**			
No. TB patients with FBG < 6.1 mmol/L	232 (100)	221 (95.3)	215 (92.7)
No. TB patients with FBG 6.1–6.9 mmol/L	_	11 (4.7)	17 (7.3)
No. TB patients with FBG ≥ 7.0 mmol/L	_	0 (0)	0 (0)
Total	232 (100)	232 (100)	232 (100)
**B: Fasting blood glucose levels at month 2 and month 6 in TB patients without DM whose fasting blood glucose was 6.1–6.9 mmol/L at the time of registration**			
No. TB patients with FBG < 6.1 mmol/L	_	9 (52.9)	10 (58.8)
No. TB patients with FBG 6.1–6.9 mmol/L	17 (100)	5 (29.4)	7 (41.2)
No. TB patients with FBG ≥ 7.0 mmol/L	_	3 (17.7)	0 (0)
Total	17 (100)	17 (100)	17 (100)
**C: Fasting blood glucose levels at month 2 and month 6 in TB patients with already diagnosed DM whose fasting blood glucose was < 7.0 mmol/L at the time of registration**			
No. TB patients with FBG < 7.0 mmol/L	8 (100)	4 (50.0)	3 (37.5)
No. TB patients with FBG 6.1–6.9 mmol/L	_	1 (12.5)	3 (37.5)
No. TB patients with FBG ≥ 7.0 mmol/L	_	3 (37.5)	2 (25.0)
Total	8 (100)	8 (100)	8 (100)
**D: Fasting blood glucose levels at month 2 and month 6 in TB patients with newly diagnosed DM whose fasting blood glucose was** ≥ **7.0 mmol/L at the time of registration**			
No. TB patients with FBG < 6.1 mmol/L	_	1 (7.7)	1 (7.7)
No. TB patients with FBG 6.1–6.9 mmol/L	_	1 (7.7)	3 (23.0)
No. TB patients with FBG ≥ 7.0 mmol/L	13 (100)	11 (84.6)	9 (69.3)
Total	13 (100)	13 (100)	13 (100)

Notes: TB = tuberculosis; DM = diabetes mellitus; FBG = fasting blood glucose. Data are presented as n (%).

Baseline characteristics of TB patients in relation to having an unstable FBG at month 2 and month 6 during anti-TB treatment are shown in Table 2. Odds on univariate analysis of a patient having an unstable FBG were caused by being HIV-positive, already known to have DM or newly diagnosed with DM, smoking cigarettes and coming to a hospital rather than to a clinic. After adjusting for possible confounding factors, odds of association with an unstable FBG were caused by being HIV positive, having DM, smoking cigarettes and those who presented to a hospital rather than a clinic.

**Table 2.** Baseline characteristics in TB patients in relation to unstable fasting blood glucose levels during the 6-month course of anti-TB treatment.

Characteristics		Total N = 270	No. (%) with unstable FBG (N = 80)	Univariate OR (95% CI)	Multivariate adjusted OR (95% CI)	*P*-value
Total		270	80 (29.6)			
Sex	Female	91	21 (23.1)	Reference		
	Male	179	59 (33.0)	1.639 (0.919–2.923)		
Age	< 20	22	5 (22.7)	Reference		
	20–59	187	47 (25.1)	1.141 (0.399–3.263)		
	≥ 60	61	28 (45.9)	4.210 (0.944–8.816)		
TB Type	Smear –	187	54 (28.9)	Reference		
	Smear +	68	21 (31.9)	1.100 (0.602–2.013)		
	EPTB	15	5 (33.3)	1.231 (0.402–3.771)		
Fever	No	183	48 (26.2)	Reference		
	Yes	87	32 (36.8)	1.636 (0.948–2.820)		
Haemoptysis	No	246	71 (28.9)	Reference		
	Yes	24	9 (37.5)	1.479 (0.619–3.534)		
HIV status	Negative	142	49 (34.5)	Reference		
	Positive	9	6 (66.7)	3.796 (0.910–15.837)	6.668 (1.245–35.706)	0.027
	Not-tested	119	25 (21.0)	0.505 (0.288–0.884)	0.640 (0.308–1.332)	0.233
Diabetes	No	249	62 (24.9)	Reference		
	Yes	21	18 (85.7)	18.097 (5.156–63.515)	17.016 (4.669–62.014)	< 0.0001
Liver diseases	No	256	74 (28.9)	Reference		
	Yes	14	6 (42.9)	1.845 (0.619–5.500)		
Smoking cigarettes/day	0	186	47 (25.3)	Reference		
	1–19	66	29 (43.9)	2.318 (1.288–4.173)	2.660 (1.374–5.150)	0.004
	≥ 20	10	4 (40.0)	1.972 (0.533–7.290)	3.010 (0.623–14.551)	0.170
	Not	8	0 (0)			
	clear					
Source of patients	TB clinic	163	32 (19.6)	Reference		
	TB hospital	107	48 (44.9)	3.331 (1.396–5.731)	2.422 (1.209–4.852)	0.013

Notes: TB = tuberculosis; DM = diabetes mellitus; FBG = fasting blood glucose; CI: confidence intervals; smear + = smear-positive pulmonary TB; smear – = smear-negative pulmonary TB; EPTB = extra-pulmonary TB.

An unstable FBG was defined as a change in fasting blood glucose of more than 1.0 mmol/L between baseline and 2 months or 6 months during the 6-month anti-TB treatment.

**Figure 1.**
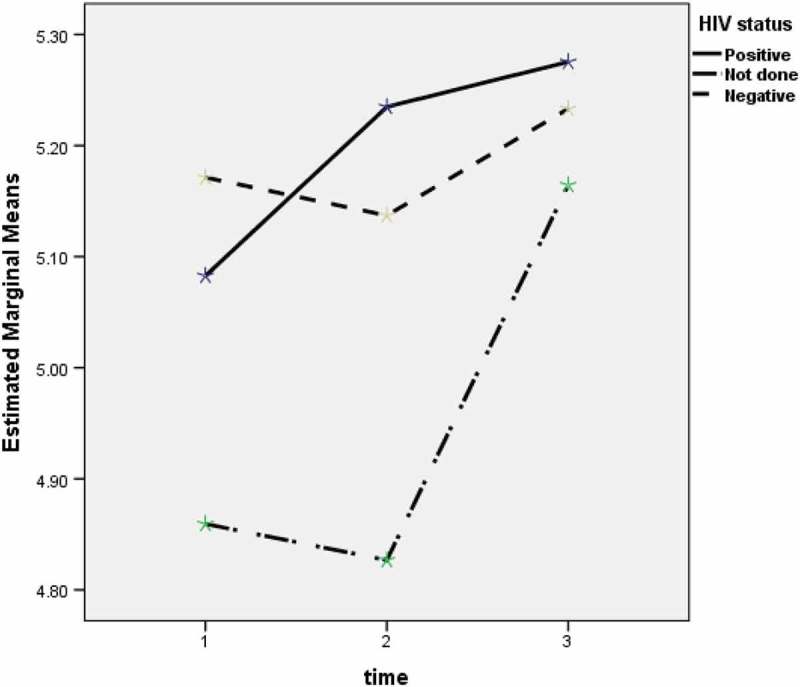
Trends of blood sugar change in TB patients who were HIV-positive, HIV-negative and HIV not-tested.

The trends in blood glucose levels during anti-TB treatment were assessed in those without DM in relation to baseline characteristics. There were no significant trends observed apart from HIV status (Figure 1): *P *= 0.164 for patients with fever and without fever, *P *= 0.133 for those with haemoptysis and without haemoptysis, *P *= 0.319 for those with liver disease and without liver disease, *P *= 0.399 for different type of TB, and *P *= 0.048 among HIV-positive, HIV-negative and HIV not-tested.

## Discussion

To our knowledge, this is the first study in China to assess changes in blood glucose levels in TB patients throughout anti-TB treatment. We conducted the study within the routine health services including both public health TB clinics and hospitals to ensure recruitment of patients with different types of TB. All patients were consecutively enrolled in the study to avoid selection bias.

The overall prevalence of DM in the recruited TB patients was 7.8%. This was lower than that observed previously when screening was done in urban areas [9], but similar to what has been observed from rural China [16]. However, it is important to note that the use of FBG alone to diagnose DM can underestimate the prevalence of this disease by as much as 50% when compared with gold standard testing using the cumbersome but more accurate oral glucose tolerance test [17]. We therefore believe that the actual prevalence of DM among our TB patients might be higher than what we have observed.

While more than half of the TB patients with already known DM had FBG less than 7.0 mmol/L during anti-TB treatment and were therefore well controlled, this was not the case with those newly diagnosed during the screening process with a high proportion continuing to have FBG at or higher than 7.0 mmol/L. This is not acceptable. Previous studies have shown that enhanced DM case management and improved blood glucose control can lead to improved TB treatment outcomes [5,18,19], and in future, DM care and high-quality treatment need to improve for our patients in order to optimize treatment outcomes.

It was reassuring that the large majority of TB patients who started anti-TB treatment with a normal FBG maintained a normal FBG or demonstrated some impaired glucose tolerance yet no patient developed DM. Similarly, of the three patients who had impaired glucose tolerance at baseline and then developed a high FBG ≥ 7.0 mmol/L at 2 months, none maintained this abnormally high FBG or developed DM by the end of treatment. This suggests that, in resource-constrained environments, a patient with FBG < 7.0 mmol/L at baseline need not be closely followed up. As early detection and treatment for DM can improve treatment outcomes for TB [7,11,18], this finding also suggests that taking one blood sample for FBG immediately after TB diagnosis would be appropriate for the majority of TB patients. These results are similar to a study on 54 Nigerian TB patients followed during treatment, which reported that nearly half had initial abnormal glucose tolerance with most reverting back to normal within 3 months of anti-TB treatment; this study suggested that glucose intolerance in pulmonary TB patients is secondary to infection and is reversible [12,19,20].

Our results in patients with impaired glucose tolerance are also similar to what has been found elsewhere [12,19–21]. Impaired glucose tolerance is an indicator for future high risk of DM, and while none of our patients in this study went on to develop DM, lifestyle counselling, health promotion and follow-up should be given to prevent or delay the onset of DM [22]. Given that patients with TB anyway are, or should be, counselled about healthy diet, avoidance of smoking, harmful use of alcohol and increasing outdoor physical activity, linking this advice at no or marginal additional cost would be beneficial as an overall public health strategy to prevent DM.

An intriguing finding that requires further study was that patients with smear-positive TB or with haemoptysis, features that tend to reflect more severe disease, were not at increased risk of having unstable blood glucose levels during anti-TB treatment. This goes against the traditional assumption and the preliminary finding of the Nigerian study that patients with more advanced or extensive disease may be more likely to have increased stress and therefore more unstable blood glucose levels during treatment.

Certain characteristics were associated with unstable blood glucose levels. Although there were only small numbers of patients with HIV-associated TB, the diagnosis of HIV was a strong risk factor. Both HIV and TB can damage the immune system and this might result in poor glycaemia regulation [23]. However, apart from noting the association, we are unable to comment further or offer any useful speculation about the reason as we have no information about stage of HIV-disease, CD4 cell counts or whether the patients were on antiretroviral therapy. One unexpected finding that requires further study was that trends in blood glucose levels against the baseline value were significantly different among those HIV-positive, HIV-negative and HIV not-tested as shown in Figure 1. The reasons are speculative, but both HIV infection itself and antiretroviral drugs have metabolic effects which may play a part. Cigarette smoking was an important risk factor and this has not been reported in previous studies. This association requires further research with possible reasons including the link between smoking and venous thromboembolism and pancreatic damage which may lead to impaired production of insulin and glycaemia regulation [24,25]. It is unclear why the association between unstable FBG and heavy smoking (smoke ≥ 20 cigarettes/day) is weak and this may in fact be due to a small number of cases. TB patients with DM, perhaps not surprisingly, had a much higher risk compared with TB patients without DM, suggesting that more attention needs to be paid to ensuring better DM control during treatment. Finally, TB patients presenting to TB hospitals also had a higher risk of unstable blood glucose compared with those presenting to TB clinics, and on discussing these findings with health workers this may be because those coming to hospital have more severe disease than those coming to clinics.

Our study has several limitations. We took three blood samples during the entire course of anti-TB treatment and therefore do not know how the FBG measurements change within and beyond month 6. In addition, blood glucose was only measured by FBG and not by glycosylated haemoglobin (HbA_1C_), which does indeed provide an index of blood glucose levels over a period of 2–3 months and is not subject to the rapid swings that can occur with FBG measurements [26]. Although only small numbers of patients with already known DM were identified, we do not know exactly what drugs were used or how they were used and thus are not able to comment on these effects on blood glucose levels. We also did not collect any information on other potentially important characteristics such as whether patients delayed accessing health service care or whether they adhered to treatment.

## Conclusion

This study shows that patients with TB who do not have DM on FBG measurement, including those with impaired glucose tolerance, do not develop DM during anti-TB treatment. However, those who are found to have newly diagnosed DM on screening in general maintain their DM status with high FBG and need to be better managed. Certain baseline characteristics such as HIV-positivity and smoking are associated with unstable blood glucose levels during anti-TB treatment and these patients need closer attention. Useful programmatic lessons can be learnt from the findings.

## Paper context

Tuberculosis patients should be screened for diabetes. However, what is the best time to take blood glucose measurements and whether one blood test is enough as tuberculosis may induce infection-related hyperglycaemia? To help answer this question, this paper reports finding from an observational cohort study to understand if blood glucose levels were stable or fluctuated during the course of anti-TB treatment in multiple settings in different provinces of China.
